# Clinically relevant mutations in the ABCG2 transporter uncovered by genetic analysis linked to erythrocyte membrane protein expression

**DOI:** 10.1038/s41598-018-25695-z

**Published:** 2018-05-10

**Authors:** Boglárka Zámbó, Zsuzsa Bartos, Orsolya Mózner, Edit Szabó, György Várady, Gyula Poór, Márton Pálinkás, Hajnalka Andrikovics, Tamás Hegedűs, László Homolya, Balázs Sarkadi

**Affiliations:** 1 Institute of Enzymology, Research Centre for Natural Sciences, Hungarian Academy of Sciences, Magyar Tudosok krt. 2, Budapest, 1117 Hungary; 2 National Institute of Rheumatology and Physiotherapy, Budapest, Hungary; 3 National Blood Service, Budapest, Hungary; 4 Department of Biophysics and Radiation Biology, Semmelweis University, Budapest, Hungary

## Abstract

The ABCG2 membrane protein is a key xeno- and endobiotic transporter, modulating the absorption and metabolism of pharmacological agents and causing multidrug resistance in cancer. ABCG2 is also involved in uric acid elimination and its impaired function is causative in gout. Analysis of ABCG2 expression in the erythrocyte membranes of healthy volunteers and gout patients showed an enrichment of lower expression levels in the patients. By genetic screening based on protein expression, we found a relatively frequent, novel ABCG2 mutation (ABCG2-M71V), which, according to cellular expression studies, causes reduced protein expression, although with preserved transporter capability. Molecular dynamics simulations indicated a stumbled dynamics of the mutant protein, while ABCG2-M71V expression *in vitro* could be corrected by therapeutically relevant small molecules. These results suggest that personalized medicine should consider this newly discovered ABCG2 mutation, and genetic analysis linked to protein expression provides a new tool to uncover clinically important mutations in membrane proteins.

## Introduction

ABCG2 belongs to the ATP-binding cassette (ABC) transporter family and plays an important role in the extrusion of wide variety of harmful compounds from our cells, protecting our body against xeno- and endobiotics, as a key participant in the so-called chemoimmunity system^1^. The ABCG2 protein is a half ABC-transporter, working as a homodimer in the cell membrane. This protein is physiologically highly expressed in the liver^2^, the intestine^3^, the blood-brain-barrier^4^, and the placenta^5^, with the role of eliminating various drugs and toxic materials, including the products of porphyrin and steroid metabolism, as well as uric acid^6,7^. Moreover, in pharmacological treatments of various diseases, the ABCG2 protein plays an important role in modulating drug absorption, distribution, metabolism, excretion and toxicity (ADME-Tox) properties^8^. In addition, ABCG2 may also be responsible for cancer multidrug resistance, as its overexpression allows tumor cells to remove chemotherapeutic agents^9–11^.

ABCG2 expression in the kidney proximal tubules and in the enterocytes has a key role in the systemic excretion of uric acid, and genome-wide association studies (GWAS) showed a significant link between gout and genetic variations in ABCG2^12–14^. A relatively common polymorphism of the *ABCG2* gene (C421A), affecting about 18–20% of people with Caucasian origin, and resulting in an ABCG2-Q141K protein variant, was shown to associate with higher serum uric acid levels and gout^14^. This ABCG2 variant has an impaired folding and cellular processing; thus, its plasma membrane expression is reduced^15^. Therefore the Q141K polymorphism may also result in alterations of pharmacokinetics of ABCG2 substrate drugs^16,17^.

In addition to the ABCG2-Q141K variant, other, less frequent SNPs or mutations may significantly affect the expression, function, or cellular trafficking of the transporter. Some of these variants have already been characterized (see Suppl. Figure 1), while currently available genome sequencing data suggest a large number (over 15,000) of SNP variants within the ABCG2 gene, and among these over 450 are missense and frameshift variants (NCBI SNP Database). Thus, it is an insurmountable task to identify variants with an effect on protein function, expression, and trafficking.

The recognition of specific genetic variants causing altered membrane protein expression could be significantly promoted by direct protein measurements in easily accessible human tissues. It has been shown recently that the erythrocyte membrane protein expression profile may serve as an information platform in this regard. In this cell membrane more than 300 membrane associated proteins are expressed^18^ (http://rbcc.hegelab.org/), including various receptors, channels, and transporters. The membrane protein expression levels depend on both genetic factors^19–21^, and regulatory alterations^22^; thus, the erythrocyte membrane protein levels can be regarded as medically useful biomarkers. Recently, we have developed a simple, fast, and reliable flow cytometry method, which allows to quantitate numerous erythrocyte membrane proteins from only a drop of blood^23^. We have also shown that reduced levels of the erythrocyte membrane ABCG2^19^ or ABCB6^20^, caused by genetic alterations, can be properly determined by using this technology.

In the present study, we have examined the expression levels of the ABCG2 protein in healthy volunteers, gout patients, as well as age-matched clinical control subjects, and mapped the genetic background of the altered ABCG2 expression levels in DNA samples. We specifically examined gout patients in this regard because of a higher *ABCG2* mutation rate expected in this disease^24^. Additionally, we also screened about 280 healthy blood donors for establishing the population-level frequency of the genetic variations found.

In addition to already described ABCG2 polymorphisms and mutations, we found a novel and relatively frequent missense mutation, ABCG2-M71V, with a low erythrocyte membrane protein expression level. We have performed detailed *in vitro* cellular expression studies for this variant, and found a strongly reduced overall and membrane ABCG2 expression. Molecular dynamics analysis of the ABCG2-M71V protein, based on the recently described atomic level structure of the ABCG2 transporter (see refs^25–28^), revealed an altered residue interaction pattern and a stumbled correlation of motions in the mutant form of the transporter.

## Results

### Quantitative ABCG2 protein expression measurements in erythrocytes, mutation studies

In these experiments we have studied blood samples of a large group (127) of normal, healthy volunteers under the age of 60 years, a group of patients (64) with clinically established hyperuricemia or gout, as well as of an age-matched control group (37) with unrelated orthopedic problems, treated at the same clinic (see Methods). In DNA samples of these groups, we performed molecular genetic analysis of the *ABCG2* gene, screening for the known, major polymorphism Q141K, and for the mutations observed by sequencing the DNA samples of donors showing lower than average ABCG2 protein expression in the erythrocyte membrane. For the location of the most important mutations and polymorphism in the protein, see the scheme in Suppl. Figure 1.

**Figure 1:**
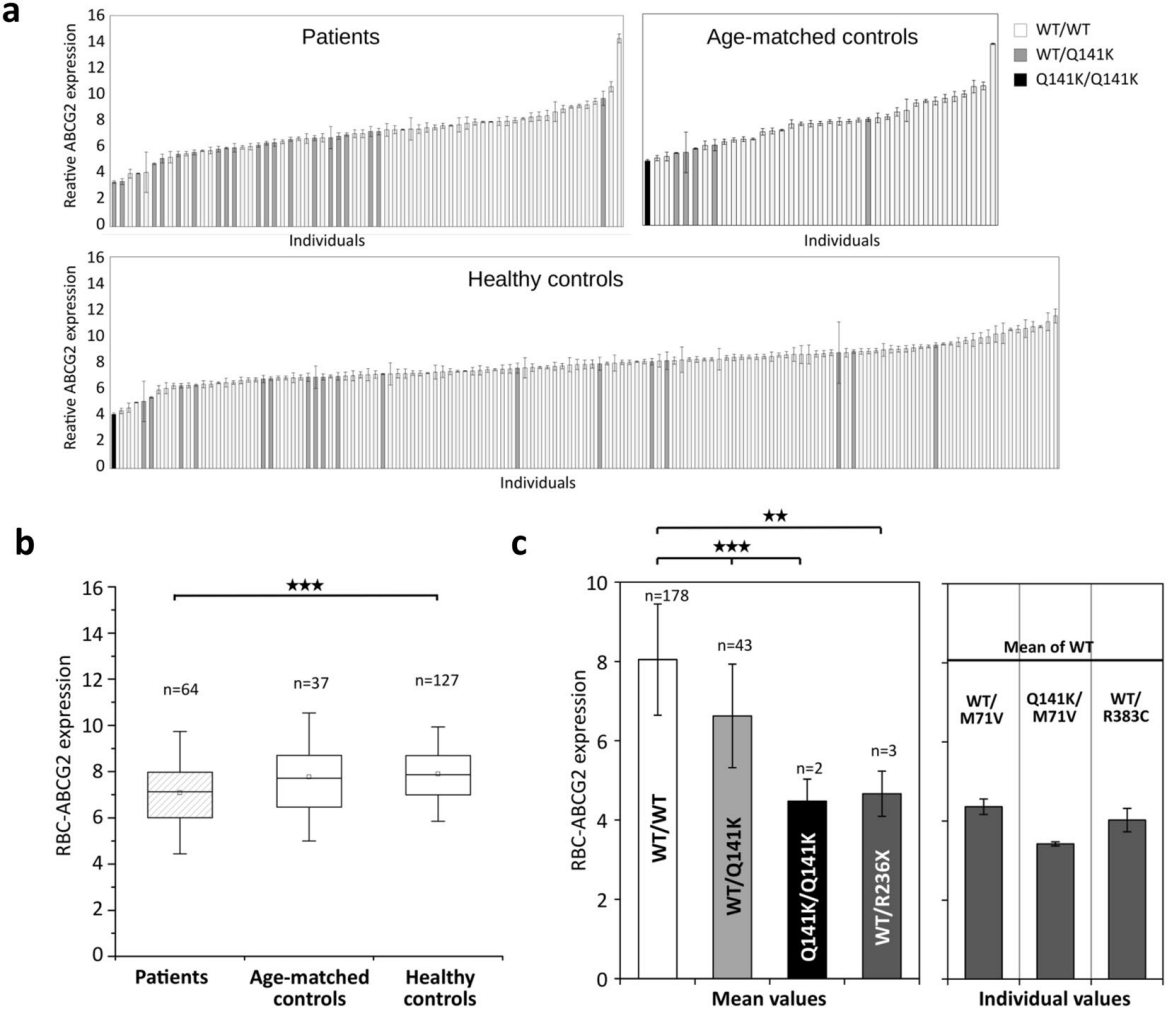
ABCG2 protein expression in the erythrocytes of gout + hyperuricemic patients and of control individuals - indication of the identified SNPs and mutations. (Panel a) Expression levels of ABCG2 in erythrocytes and the corresponding Q141K genotypes in gout patients and in control individuals. ABCG2 was detected by BXP-34 antibody and assessed by flow cytometry. The values represent the antibody labeling relative to isotype control. (Panel b) Mean values ± 25–75% quartiles (box) and ±SD (whisker) of erythrocyte ABCG2 expression levels in gout patients and in control groups (***p < 0.001). (Panel c) Left side: Mean values ± SD of erythrocyte ABCG2 expression levels in individuals carrying various polymorphisms and mutations. Right side: erythrocyte ABCG2 levels measured in individuals with identified specific mutations (mean values ± SE).

Figure 1a shows the distribution of the relative ABCG2 protein expression in the erythrocyte membranes of the cohorts examined, and the occurrence of the ABCG2-Q141K polymorphism in these individuals. As shown, this polymorphism, either in a heterozygous or in a homozygous form, was more frequent in individuals with low ABCG2 expression. Also, the occurrence of this polymorphism in the hyperuricemic + gout patients was significantly higher than that in the normal healthy control group (p < 0.01, see Table 1). Accordingly, the mean erythrocyte ABCG2 expression level was significantly lower in the patients, as compared to that seen in the healthy controls (p < 0.001, Fig. 1b). As documented in Fig. 1c, the heterozygous ABCG2-Q141K individuals had an average level of 82% ± 17%, while the homozygous individuals for this polymorphism had an average level of 56% ± 7% of the erythrocyte membrane ABCG2 expression levels, relative to that found in individuals homozygous for the wild type (Wt) transporter.

**Table 1:** Distribution of mutations and SNPs in three different cohorts: a group of patients with clinically established hyperuricemia or gout, age-matched control individuals, and normal, healthy volunteers.

	gout (n = 64)			age-matched control (n = 37)			control (n = 127)		
	homoz.	heteroz.	wild type	homoz.	heteroz.	wild type	homoz.	heteroz.	wild type
Q141K	0	21	43	1	5	31	1	18	108
M71V	0	1	63	0	0	37	0	1	126
R236X	0	2	62	0	0	37	0	1	126
R383C	0	1	63	0	0	37	0	0	127

In several patients and volunteers, we found very low (about 50% of average) ABCG2 erythrocyte membrane expression levels. In these cases, we performed a sequencing of the *ABCG2* gene to explore the potential genetic background of this low level ABCG2 membrane expression. As shown in Fig. 1c, among these individuals we found three heterozygous individuals with an already described nonsense mutation R236X, thus reinforcing the role of this mutation in causing a decreased ABCG2 expression^19^. In addition, we found one heterozygous individual carrying a mutation of ABCG2-R383C (Table 1), which has been shown to be a folding mutant in *in vitro* expression experiments^29^, while its effect has not been explored in human samples.

In addition to these known variants, among the individuals with lower ABCG2 expression, we found a mutation, 211 A > G (rs148475733), leading to the ABCG2-M71V variant. This mutation caused an about 50% reduction in ABCG2 expression in heterozygotes, and even a lower level of ABCG2 expression in an individual carrying both this mutation and the Q141K polymorphism (see Fig. 1c). Western blot analyses of red blood cell membranes further supported our flow cytometry-based findings about reduced RBC protein levels of ABCG2-M71V (Suppl. Figure 2). Since we found one heterozygous individual for this mutation among the gout patients and one among the healthy volunteer group, we screened the DNA samples of a larger cohort of healthy individuals for the prevalence of the ABCG2-M71V variant. To explore the frequency of the ABCG2-M71V variant in the normal population, we screened for the above-mentioned polymorphisms/mutations in DNA samples of the previously studied group of healthy individuals and an additional cohort of 278 healthy, normal blood donors (Table 2). According to these data, the heterozygous prevalence of the ABCG2-M71V variant, causing low ABCG2 expression, is relatively frequent (expected to be about 1%) in the general Hungarian (mostly of Caucasian origin) population.

**Table 2:** The occurrence of the identified mutations and SNPs in the normal, healthy population.

	Total normal controls (n = 405)				
	homoz.	heteroz.	wild type	MAF	HW(Chi^2^)
Q141K	5	68	332	0.0963	0.8941
M71V	0	4	401	0.0049	0.9950
R236X	0	3	402	0.0037	0.9972
R383C	0	0	405	n.d.	n.d.

MAF (minor allele frequency), and HW (Hardy-Weinberg) equilibrium values were calculated from the merged control group (n = 405) consisting of the healthy volunteer group shown in Fig. 1/Table 1, and a cohort of additional 278 healthy blood donors.

### *In vitro* expression and functional studies of the ABCG2-M71V variant in mammalian cell lines

In the following *in vitro* experiments, we aimed to find out the expression and functional properties of the ABCG2-M71V variant in cell lines, causing lower *in vivo* erythrocyte membrane expression levels. Therefore, we expressed three different vector constructs (see Methods and Suppl. Figure 3), coding for this variant, in three different mammalian cell lines. The first construct was an expression plasmid, in which the transient expression of the ABCG2 cDNA was driven by a CMV promoter; while the second plasmid contained a cDNA coding for GFP-ABCG2, driven by the same promoter. As we have shown earlier, the N-terminally GFP-tagged ABCG2 protein is properly processed in various cell types and is fully functional when membrane inserted, thus this construct allows a direct, fluorescence-based visualization of the transporter and its function^30^. In the third construct, in addition to ABCG2, we inserted an IRES-GFP, allowing a direct estimation of the transfection efficiency and the flow cytometry-based selection of the protein expressing cells. For the present studies we preferentially used this latter, well-controlled transient expression system, providing faster and more reliable comparative protein expression results than a stable expression system.

For the ABCG2 expression studies we have selected cell lines in which the expression of endogenous ABCG2 is very low, and these were the human embryonic kidney (HEK) 293 cells, the human HeLa cancer cells, and the dog kidney, polarizable MDCKII cells. In addition, for functional studies, we have also expressed both the wild type and the ABCG2-M71V variants in the baculovirus/Sf9 cell protein expression system. It has been shown in several studies that these insect cells process potential folding/trafficking mutants of human membrane proteins (e.g., CFTR or ABCG2) much better than various mammalian cell lines^31,32^, thus allow functional studies even for these mutant variants.

**Figure 2:**
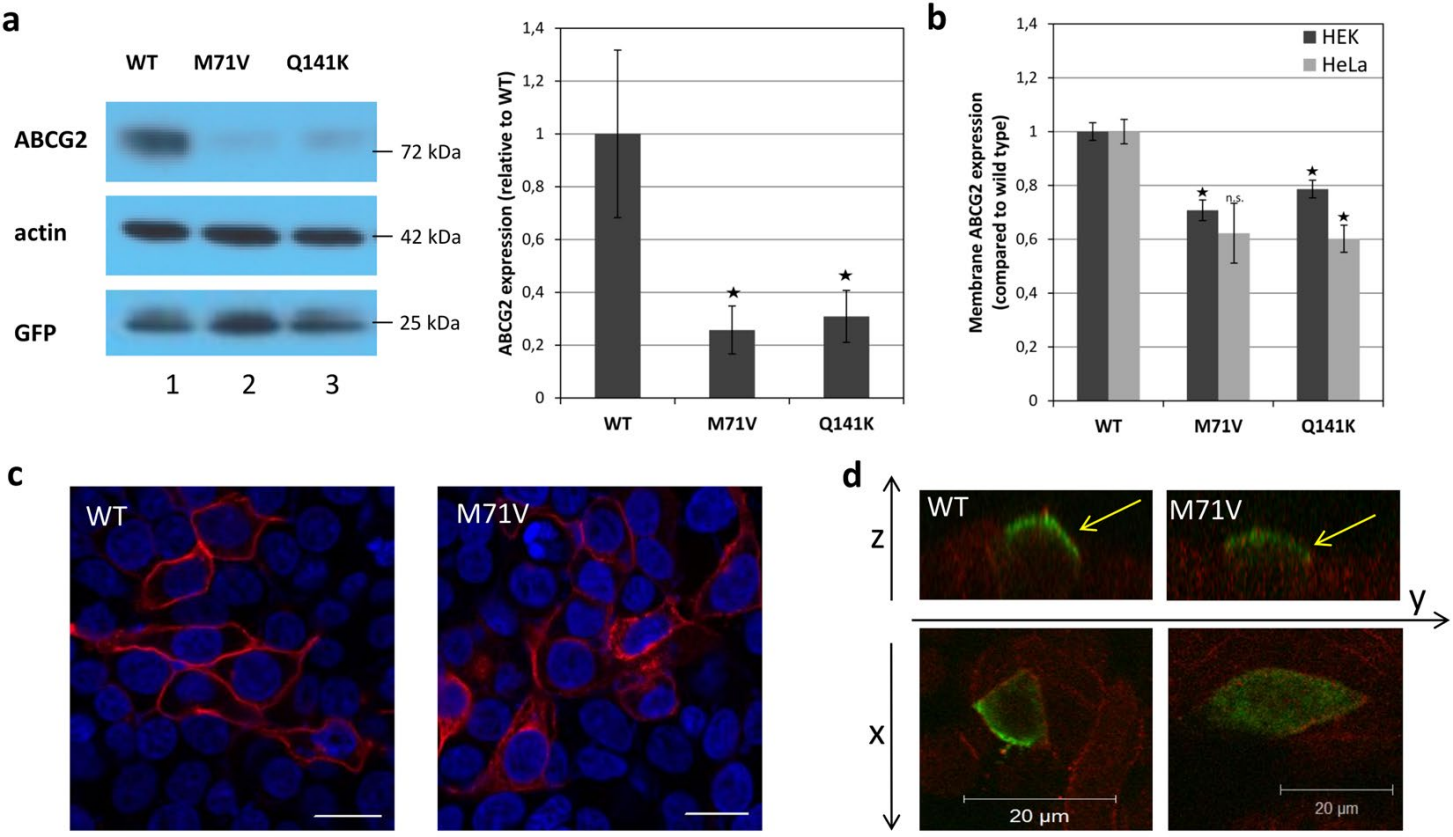
Determination of the expression and localization of ABCG2 variants in cellular systems *in vitro*. (Panel a) Expression of ABCG2 variants in HEK293 cells, detected by immunoblotting. Left Panel – immunoblot, Right Panel - expression levels of the wild type ABCG2, the ABCG2-M71V and the ABCG2-Q141K variants, normalized to beta-actin and presented as relative expression, compared to the ABCG2-Wt. Bars represents the mean relative expression (±SEM) from triplicate Western blots (Student’s t-test, *p < 0.05). The different proteins examined in the Western blot were developed by the respective antibodies and cropped from the indicated parts of the same gel and blot. (Panel b) Cell surface membrane expression of ABCG2 variants in HEK293 and HeLa cells, detected by the 5D3 antibody reacting with an extracellular epitope. Flow cytometry measurements (±SEM; Student’s t-test, *p < 0.05, **p < 0.01, compared to the expression of the wild type ABCG2 in the same cell type). (Panel c) ABCG2 and ABCG2-M71V protein expression visualized by confocal microscopy in transfected HEK293 cells. ABCG2 is labeled with BXP-21 antibody (red), and nuclei are stained with DAPI (blue). Scale bars represent 20 µm. (Panel d) Localization of ABCG2 and ABCG2-M71V protein as observed by confocal microscopy in transfected and polarized MDCKII cells. ABCG2 is stained with the BXP-21 antibody (green), and the basolateral membrane marker Na^+^/K^+^ ATPase is stained with a specific antibody (red). Yellow arrows point to the apical membrane.

Figure 2a documents the transient expression of the IRES-ABCG2 protein variants in HEK cells after 2 days, as measured by Western blotting. The ABCG2-Wt protein was properly expressed (and appeared to be fully glycosylated), with the expected relative molecular mass (72 kDa), while the ABCG2 expression in the mock control was not detectable (see Supplementary Figure 8). In contrast, the expression of the ABCG2-M71V variant was much lower, although its glycosylation was preserved. The average expression levels of the M71V variants, as compared to the Wt ABCG2, were between 20–40%, when examined in three independent experiments. Since we did not see differences between the GFP levels in the cells expressing the Wt and the variants of ABCG2 from the IRES containing vectors, we did not expect differences between the mRNA levels of the ABCG2 variants (for the IRES containing vector see Suppl. Figure 3). Still, we have performed additional qPCR studies, which showed similar ABCG2 mRNA expression levels in these cells, thus reinforcing this notion (Suppl. Figure 4). When using the other ABCG2 expression vectors for ABCG2 expression studies, Western blotting showed similar expression differences between the wild type versus the M71V variant of the ABCG2 protein (see Suppl. Figure 5). In the case of the GFP-fused ABCG2-M71V protein, the expression levels, as compared to the GFP-ABCG2(Wt) protein, were even lower in both model cell lines – in HEK cells the expression of the variant was below 10%, in the HeLa cells it was undetectable (Suppl. Figure 5).

For examining the cell membrane localization of the ABCG2-M71V variant, we performed cell surface labeling studies, by using the 5D3 monoclonal antibody, recognizing an extracellular epitope of the ABCG2 protein^33^. In these studies, we used the ABCG2-IRES-GFP transient expression transfection vector, in order to be able to select GFP- (and thus ABCG2-) expressing cells by flow cytometry gating, while a potential effect of the GFP tag on ABCG2 expression was excluded. For comparison, we also examined in these experiments cells transiently expressing the Q141K variant of the ABCG2 protein. As shown in Fig. 2b, the cell surface expression of the ABCG2-M71V variant in both HEK 293 and HeLa cells was about 60–70% of that of the Wt ABCG2 (in 3 independent experiments). Thus, lower expression corresponded to a lower cell surface appearance of the ABCG2-M71V variant, and these results were reinforced by further confocal microscopy studies (see below). The ABCG2-Q141K variant also showed lower membrane expression, as expected from previous studies.

Figure 2c presents a representative confocal microscopy image of the HEK 293 cells transiently expressing either the wild type ABCG2 or the ABCG2-M71V variant. The M71V protein in these cells clearly shows partial plasma membrane localization but the intracellular accumulation of the protein is also visible.

In the following experiments we have expressed the wild type ABCG2 and the ABCG2-M71V variant in polarized MDCKII cells. In these polarized cells immunostaining with BXP-21 monoclonal antibody allowed the examination of the apical/basal localization of the overexpressed human proteins. As shown in Fig. 2d, we found that both the wild type ABCG2 and the ABCG2-M71V variant showed a proper, apical localization in these cells. Thus, the apically oriented trafficking of the mutant seems to be preserved in this system.

**Figure 3:**
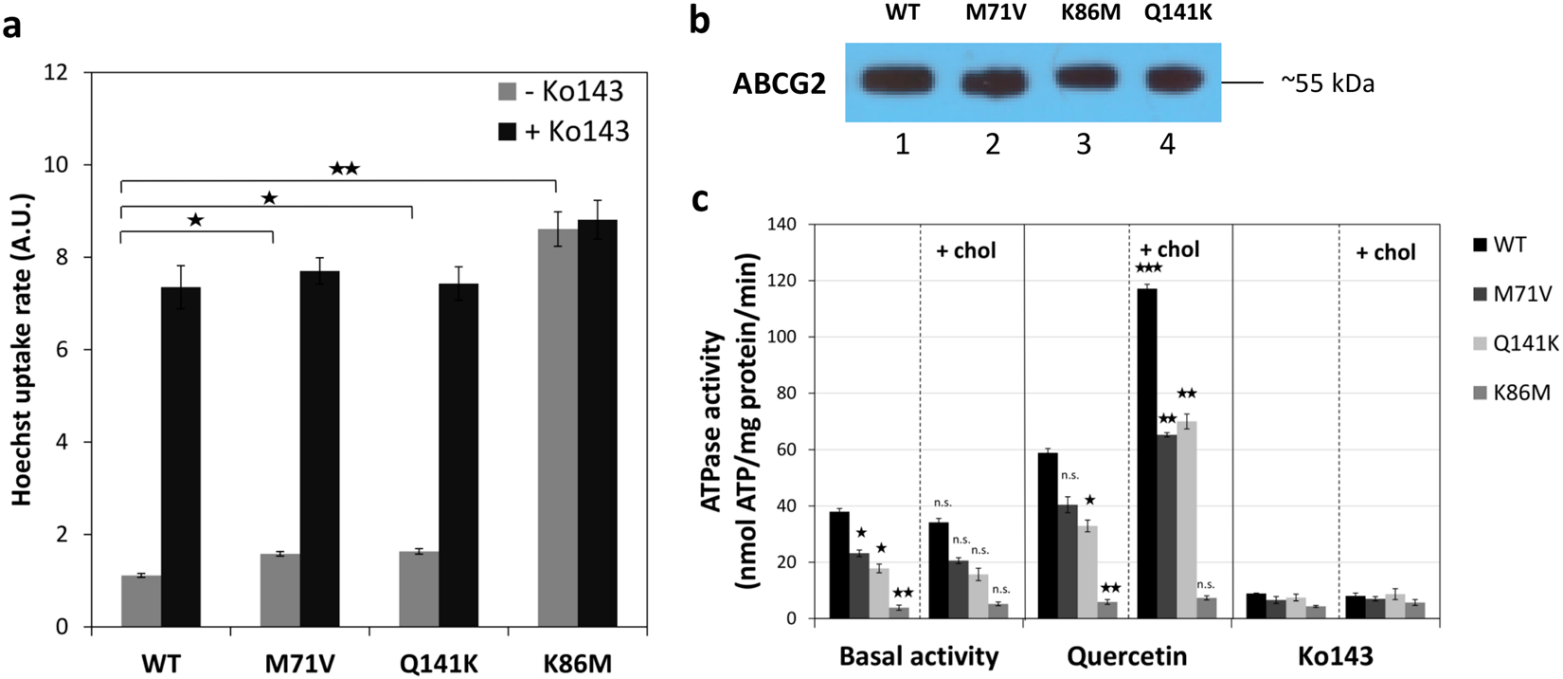
Functional analysis of ABCG2 and its variants in cellular expression systems. (Panel a) ABCG2-dependent Hst dye extrusion in HEK 293 cells. The initial slopes were determined from the kinetic curves of cellular dye accumulation assessed by flow cytometry in the absence and presence of Ko143, a specific inhibitor of ABCG2 inhibitor (n = 3, ±SE, significance determined by Student’s t-test). (Panel b) Expression levels of ABCG2 variants (Wt, M71V, Q141K, and K86M) in isolated Sf9 membranes, as measured by Western blotting, using the BXP-21 antibody. The different proteins examined in the Western blot were developed by the respective antibodies and cropped from the indicated parts of the same gel and blot. (Panel c) ABCG2-ATPase activity in isolated Sf9 membranes, expressing the ABCG2 (Wt, M71V, Q141K, and K86M) variants. Vanadate-sensitive ATPase activity in the absence or presence of membrane cholesterol was measured, either in the absence of additional compounds (basal activity), in the presence of 10 µM quercetin, providing maximum drug-stimulated activity, or in the presence of 5 µM Ko143. (±SEM, significant differences were determined with Student’s t-test) In the left panels (without cholesterol), the mutant variants were compared to wild type, whereas in the right panels (with cholesterol) cholesterol-treated membrane preparations were compared to the corresponding untreated samples. *p < 0.05, **p < 0.01, ***p < 0.001.

The application of the ABCG2-IRES-GFP transient expression system allowed proper assessment of ABCG2 function, by performing flow cytometry transport measurements for the wild type ABCG2 and the ABCG2-M71V variants. In this case we measured the cellular uptake of Hoechst 33342 (Hst), a widely applied, transported fluorescent substrate of ABCG2, in ABCG2-expressing cells selected on the basis of GFP expression. In Fig. 3a, we show the Hst transport activities in the GFP-positive HEK 293 cells.

According to these data, the cell surface expression of the ABCG2-M71V and the ABCG2-Q141K variants were significantly lower than that of the wild type ABCG2, and the observed transport activity corresponded to these lower membrane expression levels (the ABCG2-K86M catalytic mutant variant had no transport activity). These data indicate that the ABCG2-M71V variant is an expression mutant, but probably with a preserved transport activity, if reaching the cell surface membrane.

In order to examine the function of the ABCG2-M71V variant, we also expressed this protein and the wild type ABCG2 in the baculovirus-Sf9 cell system, and measured ABCG2-ATPase activity in isolated membranes. It has been shown earlier that human ABC protein expression in insect cells, although in an underglycosylated form with lower apparent molecular masses, allows functional studies even in the case of folding/trafficking mutant variants, poorly expressed in mammalian cell lines^31,32^. Also, the basal and drug-stimulated ABCG2-ATPase activities have been documented to reflect the function of the transporter^32^.

As documented in Fig. 3b, the membrane expression levels of the ABCG2-Wt, the ABCG2-M71V, the ABCG2-Q141K, and the ABCG2-K86M variants in Sf9 cells were similar, estimated by Western blotting. Regarding ABCG2-dependent membrane ATPase activity (specifically inhibited by Ko143), either without added drugs, or in the presence of the ATPase-stimulating substrate, quercetin, both the M71V and Q141K ABCG2 variants exhibited substantial ATPase activities, although somewhat lower than those of the wild type ABCG2 (Fig. 3c). The ABCG2-K86M catalytic mutant had practically no ATPase activity. As documented earlier^34^, cholesterol loading of the Sf9 cell membranes greatly increased the specific ATPase activity of the wild type ABCG2 and, as shown in Fig. 3c, a similar increase was observed for the M71V and Q141K variants. These experiments indicate a slightly lower but preserved catalytic activity of the ABCG2-M71V protein, and a similar modulation by membrane cholesterol as that for the wild type protein. In addition, we examined the effect of uric acid, an ABCG2 substrate related to gout development, on the ABCG2-ATPase activity in isolated insect cell membranes. As shown in Supplementary Figure 6, uric acid significantly stimulated the ATPase activity of both the wild type and the M71V or the Q141K variants of ABCG2, indicating again a preserved function of these variants.

### In silico structural and molecular dynamics studies of the ABCG2-M71V variant

**Figure 4:**
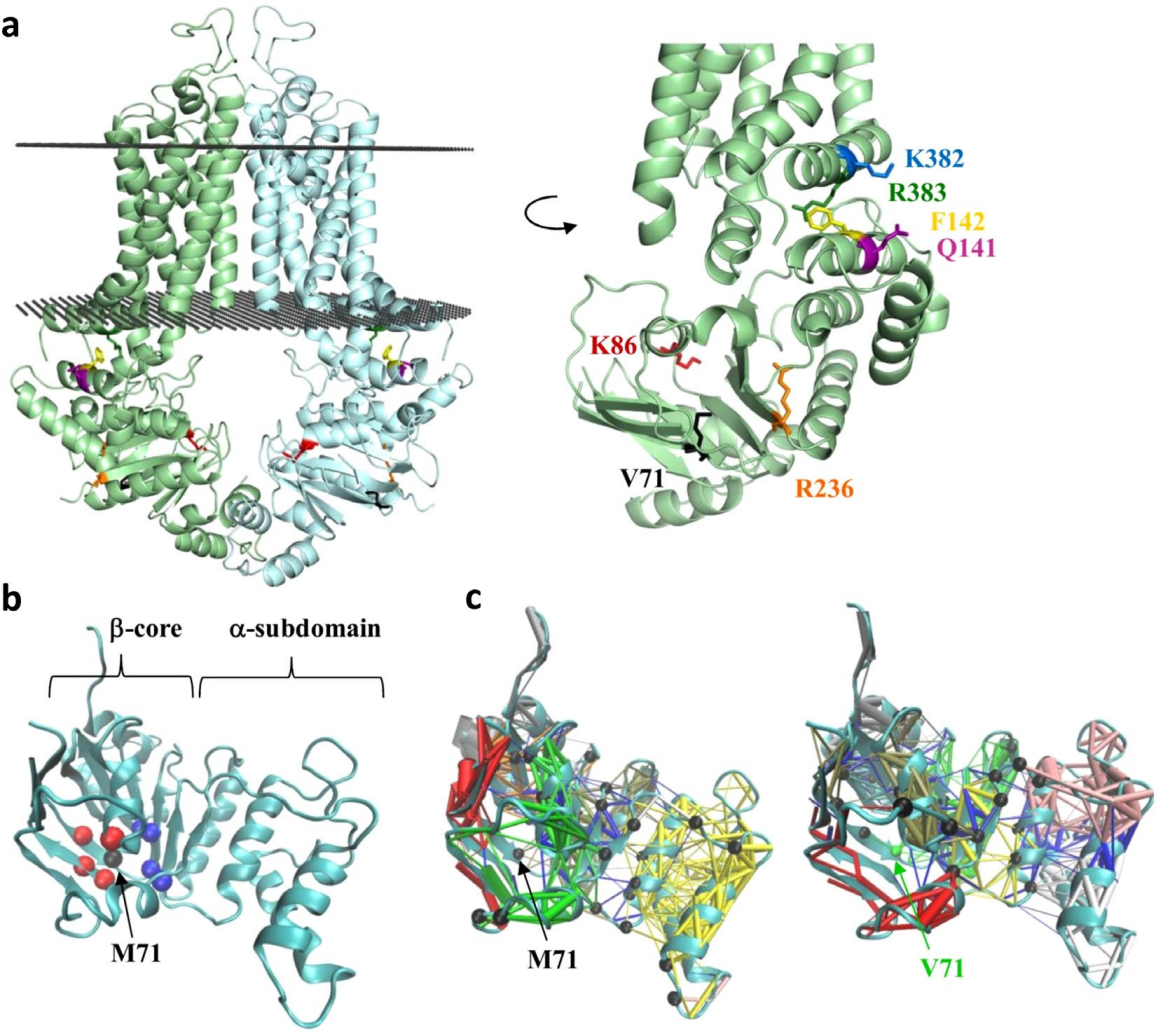
Location of the mutations in the ABCG2 structure – analysis of molecular dynamics. (Panel a) Left side: model of the ABCG2 dimer in the membrane. Membrane insertion of the ABCG2 structure was determined by the OPM server. Right side: NBD and NBD/TMD interfaces are magnified and selected positions for mutations are highlighted. Black dots: bilayer boundaries; green and cyan: ABCG2 protomers. (Panel b) The most frequent interactions of M71 throughout MD simulation trajectories are highlighted. Black: M71; blue: residues, the interaction of which did not change in M71V as compared to Wt; red: residues exhibiting increased interactions with M71 in the mutant as compared to the Wt. (Panel c) Dynamic communities of residues determined by the analysis of a network, generated on the basis of the correlation of motions, are labeled with different colors. Black spheres represent critical links between communities.

Based on the recently published atomic level structure^25^ and our homology model^35^ of the homodimeric ABCG2 protein, the M71V mutation is localized in the ATP-binding β-core subdomain of the NBDs (see Fig. 4a and b). In order to understand the effect of this mutation on the structure and dynamics of ABCG2, we performed molecular dynamics simulations using the wild type and the mutant NBD structures. Val at position 71 was found to exhibit an increased number of interactions with some neighboring amino acids (Fig. 4b, Suppl. Figure 7), related to the smaller side chain and increased side chain dynamics of this residue, as compared to those of Met. Some of the main residue interactions of 71V did not change (residues 76, 206, 237, and 239, located on the opposite site of the β-core subdomain, relative to amino acid 71, as shown by blue spheres in Fig. 4b), while contact frequency of 71V with other residues increased (residues 33, 34, 69, and 93, red spheres in Fig. 4b). In order to understand the larger scale effect of these differences, we performed the analysis of a network, built on the basis of correlated motions of residues. We found that identified communities in the Wt domain are well-related to structural elements, e.g., the residues in the α-subdomain form a community (Fig. 4c/1, yellow). Importantly, one of the critical residues (Fig. 4c/1, black spheres), which communicate allosteric changes between the communities, is M71. As a result of the M71V mutation, the network and community of residues with correlated motions are altered dramatically. Communities became unrelated to structural elements (e.g. the α-subdomain is split to different dynamic communities and critical residues) and the intradomain allosteric pathways were altered. These observations, based on events occurring in the nanosecond time scale, suggest that the ABCG2-M71V suffers from imbalanced motions that likely effect events happening on longer time scales, including alterations in the folding pathway and a decreased processing of the protein. In addition, this type of altered dynamics may also impair domain-domain assembly as observed in the case of CFTR mutants^36,37^.

### Modulation of the expression of the ABCG2 variants by 4-PBA and colchicine; effects of proteasome inhibitors

In the following experiments we examined the potential modulatory effects of 4-phenylbutyrate (4-PBA) and colchicine on the expression of the ABCG2 variants, as an increased expression of ABC membrane proteins was reported by these compounds^38,39^. Although the effects of these agents on transcription and expression of cellular proteins is relatively non-specific, these relatively non-toxic molecules have been examined both in experimental conditions^15,38^ and in clinical trials^40,41^, in several membrane protein related diseases. We have examined if the membrane localization of the wild type and variant ABCG2 proteins could be improved by these treatments.

**Figure 5:**
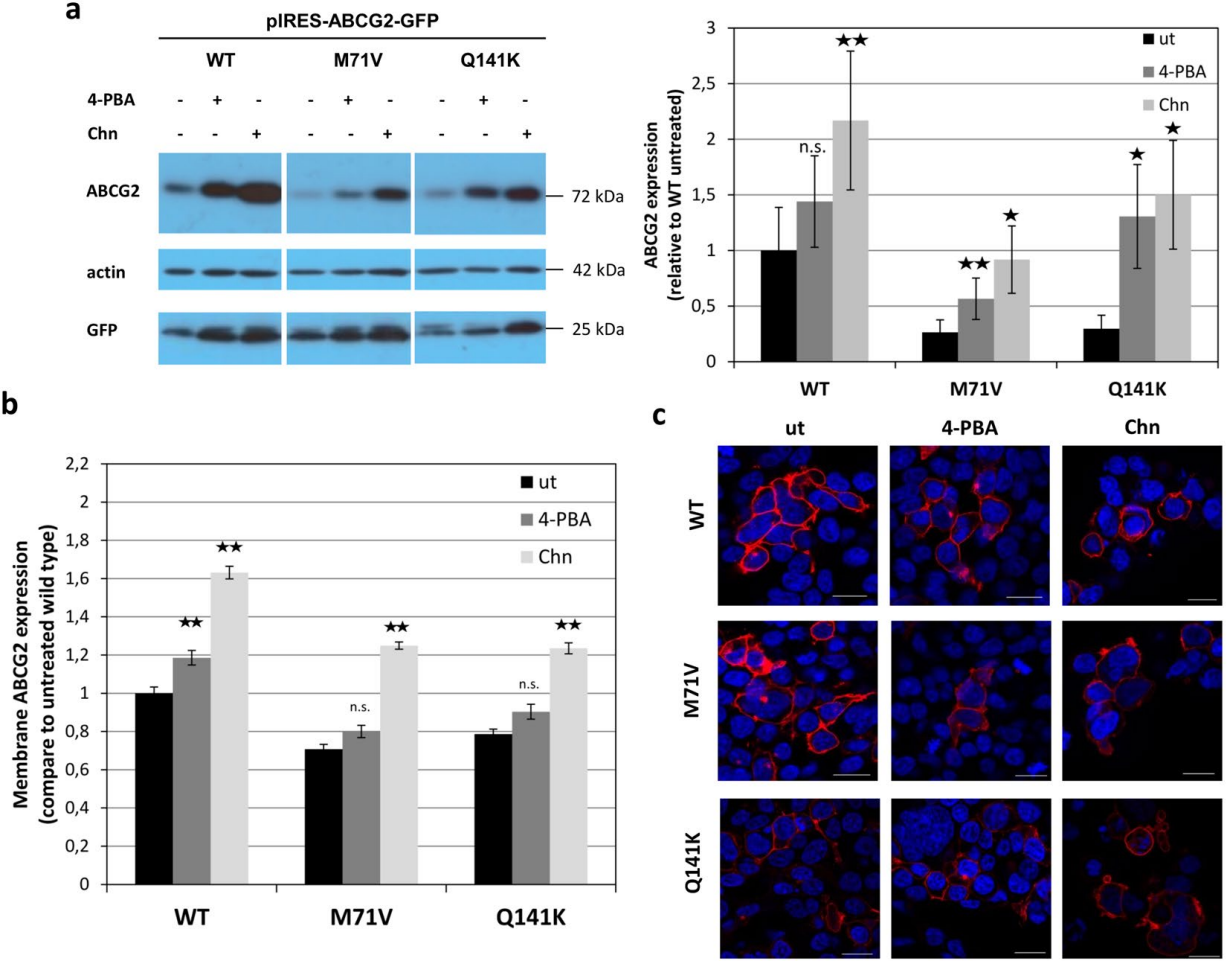
Impact of 4-PBA and colchicine on the expression and membrane localization of the ABCG2 wild type and the ABCG2-M71V protein in HEK293 cells. (Panel a) Total ABCG2 expression determined by immunoblotting in HEK293 cells transfected with the ABCG2-IRES-GFP vector, following a 24-hour exposure to 1 mM 4-phenylbutyrate (4-PBA) or 1 µM colchicine (Chn). Left side: Western blot. The different proteins examined in the Western blot were developed by the respective antibodies and cropped from the indicated parts of the same gel and blot. Right side: the results are presented as relative expression levels, compared to that of the untreated ABCG2-Wt. Bars represent the mean relative expression (±SEM) values from three independent experiments. (Panel b) Surface localized ABCG2 protein expressions in the HEK293 cells, treated as above, were detected by 5D3 antibody binding determined in flow cytometry (±SEM, significance assessed by Student’s t-test). (Panel c) ABCG2 expression in the HEK293 cells treated as above, determined by Bxp-21 antibody labeling (red) following a 24-hour 4-PBA or colchicine treatment. Nuclei are stained blue by DAPI. Scale bars represent 20 µm. (ut: untreated, Chn: colchicine-treated).

As documented in Fig. 5a, both the addition of 4-PBA (1 mM) and colchicine (1 µM) after 24 hours significantly increased the overall expression of all the ABCG2-Wt, the ABCG2-M71V, and the ABCG2-Q141K proteins in HEK cells (for a full blot see Suppl. Figure 8). In addition, the cell surface localization of the ABCG2 variants, as determined by 5D3 antibody binding, also significantly increased (Fig. 5b). In confocal microscopy studies an increased expression and membrane localization was also observed in the 4-PBA- or colchicine-treated HEK cells (Fig. 5c). The effects of 4-PBA and colchicine was relatively non-specific, as GFP expression was also increased in the treated cells (see Fig. 5). Still, the detailed analysis of these changes (Suppl. Figure 9) showed that while ABCG2 expression, especially in the case of the variants, was greatly elevated, the increase in GFP expression was much smaller, and that in the expression of actin was negligible.

In order to examine whether the reduced protein levels of the polymorphic ABCG2 variants were due to enhanced proteasomal degradation, we investigated the effects of proteasome inhibitors on the ABCG2 expression levels. We found that 5 nM bortezomib or 10 µM MG132 (strongly inhibiting proteasome activity in a relevant *in vitro* assay) did not increase the protein levels of the ABCG2-M71V and ABCG2-Q141K in transiently transfected HEK cells (Suppl. Figure 10). This observation indicates that proteasomal degradation has no crucial role in the reduced expression of the studied variants under these conditions.

## Discussion

In our earlier work we have set up a system to quantitate membrane protein expression levels in the human erythrocytes, with the aim of finding biomarkers for diseases in which membrane proteins play a key role^22,23^. Based on antibody-based quantitative flow cytometry studies, corroborated by mass spectrometry measurements^42,43^, a basic methodology panel^23^ and a corresponding database^18^ (http://rbcc.hegelab.org/) for erythrocyte membrane protein expression have been generated. According to these studies, currently over 220 membrane proteins show verified expression in the human erythrocyte membrane; and among these we established quantitative flow cytometry determination technology for over 15 clinically important membrane proteins, including receptors, channels, and transporters (e.g., INSR, GLUT-1, ABCA1, ABCB6, ABCG2, URAT-1). We have already performed a clinically oriented membrane transporter expression study in Alzheimer’s disease^22^, and currently working on a larger study related to type 2 diabetes (unpublished).

Recently, we have initiated a clinically oriented project for analysing membrane protein expression alterations in gout and hyperuricemic patients. Based on GWA studies, mutations or polymorphisms in several key membrane proteins involved in urate transport, including SLC22A11/OAT4, SLC22A12/URAT1, SLC17A1/NPT, and especially in SLC2A9/GLUT9 and ABCG2, show strong association with gout^44–46^. Since the erythrocyte membranes express relatively high levels of the ABCG2 protein (providing the blood group antigen Jr), we have started these investigations by the quantitative measurement of the ABCG2 multidrug transporter protein in gout and hyperuricemic patients, as well as in age-matched control subjects. We have also measured the expression levels of the ABCG2 protein in the erythrocytes of a cohort of healthy volunteers and performed a detailed molecular genetic characterization of *ABCG2* in all these individuals, supplemented with the examination of the DNA samples of 278 healthy blood donors.

As documented, individuals with low level erythrocyte ABCG2 expression were found with an increased occurrence among the gout/hyperuricemic patients, as compared to the control individuals. When examining the genetic background in the corresponding DNA samples, in accordance with data in the literature^15^, we found an increased occurrence of the 421 C > A (ABCG2-Q141K) variant in the patients, correlating with lower erythrocyte expression (see^19^). For the individuals showing significantly lower than average erythrocyte ABCG2 expression, we performed ABCG2 sequencing. Among these individuals we found previously described mutations leading to impaired ABCG2 expression – these were three heterozygous individuals carrying the nonsense mutation R236X^17,19^, and one heterozygous individual carrying a missense mutation, R383C. This latter mutation, although not examined as yet in human samples, has been already characterized in detailed *in vitro* expression studies, and was found to result in impaired protein folding, abrogated glycosylation and membrane surface expression in mammalian cells, as well as a loss of transport activity in an Sf9-baculovirus expression system^29^.

In addition to these mutations, we found two heterozygous individuals carrying a so far unknown *ABCG2* missense mutation, 211 A > G, resulting in the ABCG2-M71V protein with lower expression in the erythrocytes of heterozygous individuals. Western blot measurements, although less quantitative, also supported our findings about reduced ABCG2 protein levels in the RBC membrane (see Suppl. Figure 2). When screening the DNA samples of the cohort of the healthy volunteers (160), and DNA obtained form an additional 278 blood donors, altogether we found five heterozygous individuals carrying the R236X, one individual with the R383C, and 5 individuals with the M71V mutation. Considering only the healthy volunteers, representing a general population in Hungary, the number of M71V mutations corresponds to MAF (minor allele frequency) values of about 0.5%. Thus, we observed a relatively frequent new ABCG2 mutant variant, M71V, leading to decreased erythrocyte membrane protein expression. According to databases, this mutation occurs almost only in the Caucasian population. The minor allele frequency in this population, determined by the 1,000 Genome Project, was 0.0009, whereas no carriers of M71V were found in other regions. The rarity of this mutation hinders the determination of its allele frequency in populations other than Caucasian. In our study, we did not find individuals carrying the Q126X mutation, described to be present in Asian populations (see ref.^12^).

SNPs or mutations in the ABCG2 gene may variably affect the expression, function, or cellular trafficking of the transporter, and lower erythrocyte membrane levels may point to impaired expression, including translation, folding or processing. A common SNP, 34 G > A, resulting in ABCG2-V12M, and present in about 10% of the population with Caucasian origin, did not result in lower erythrocyte membrane expression^19^ and experimental studies showed that this missense variant had no significant effect either on the function or the localization of ABCG2^19,47^. In contrast, in previous studies we showed that the Q141K variant, associated with gout and showing reduced *in vitro* expression^15^, as well as naturally occurring stop or frameshift mutations, caused lower erythrocyte ABCG2 expression levels^19^.

In order to explore the characteristics of the ABCG2-M71V variant found in this study, we have performed detailed *in vitro* cellular expression and *in silico* modeling studies. In various mammalian cell models, including HEK, HeLa and MDCKII cells, we found significantly lower overall expression of the ABCG2-M71V variant as compared to that of the wild type transporter. Although the expression levels were low, ABCG2 glycosylation was fully retained, and the expressed protein reached the plasma membrane – in the polarized MDCKII cells even the apical membrane localization was preserved. Moreover, the ABCG2-M71V variant showed a preserved activity when expressed either in the mammalian cells, or the baculovirus-Sf9 insect cell system. In this latter case, the ATPase activity of this variant was somewhat lower than that of the wild type protein, but drug-stimulation and cholesterol-dependence of this activity were similar to that seen in the wild type protein. According to these results, the M71V mutant has reduced expression levels and partially preserved activity, similar to the Q141K polymorphic variant, but the M71V mutation causes even stronger reduction in expression, both *in vitro* and *in vivo*, as observed in the human erythrocyte membrane.

In these experiments we found that the GFP-tagged version of the M71V protein was practically not expressed at all – probably any expression problem is accelerated in the case of this fusion construct. In addition, molecular dynamics analysis of the ABCG2-M71V protein, based on recently published structural models^25,35^, revealed an altered interaction pattern of the mutant residue and changes in intradomain allosteric communication pathways.

Our findings suggest that the M71V mutant has a major cellular expression problem, which may cause low *in vivo* expression levels and substantial alterations in the drug metabolism and uric acid excretion in human subjects. In heterozygous individuals this may result in more pronounced effects than the Q141K polymorphism, already shown to promote gout development, while in homozygous individuals a folding mutant may produce a Jr- blood group property^48,49^, resulting in major transfusion or child-bearing problems^50^. Since homozygous ABCG2-M71V individuals are expected to occur only in 1 among 10,000 individuals of Caucasian origin, further population wide studies are required to clarify these potential effects. Further detailed clinical and molecular genetic studies are required to explore the effects of this mutation in gout, in ADME-tox properties of ABCG2 substrate drugs, e.g., statins, and in the treatment of drug resistant cancer cells (see^46^).

As shown above, although with an impaired expression, the remaining M71V protein still can reach the plasma membrane and retain function, thus, a correction of the expression level of this mutant may result in proper membrane localization and transport. Corrector molecules promoting membrane expression of at least partially functional mutant variants of the ABCC7/CFTR protein already reached clinical application^51^.

In order to explore this possibility, we have examined the effects of two potential corrector molecules, 4-PBA and colchicine. These compounds, which have been reported to increase membrane protein processing for ABCG2^38^, ABCC6^39^, and ABCB11^40^, are FDA and EMA approved pharmacological agents already used at the clinic^51–55^. Moreover, colchicine is efficiently used in gout attacks, presumably because of reducing the local inflammatory response^53^. Here we found that both compounds significantly improved the expression of the wild type ABCG2, the ABCG2-M71V, as well as the ABCG2-Q141K protein. The expression level and the transport capacity of these latter two variants in the corrector-treated cells reached the level of that of the Wt protein under control conditions. Although 4-PBA and colchicine are relatively non-specific agents, these relatively non-toxic molecules may have clinical value in the treatment of gout, and in fact, colchicine may at least partially evoke its beneficial effect in gout by increasing ABCG2 expression. 4-PBA and colchicine are currently used to treat several diseases, including the correction of trafficking problems e.g., of BSEP (ABCB11) in the liver disease PFIC2^55^, and CFTR in cystic fibrosis (see^41^ and www.clinicaltrials.gov). Also, our data indicate that by accelerating protein expression, the ABCG2-M71V protein defect can be corrected, as the protein itself can be membrane inserted and functional. Further drug development efforts for finding more specific agents for an allele-specific therapy in gout patients may be stimulated by these basic findings. The potential correlation of the effect of colchicine and the presence of the M71V and/or the Q141K variants in gout patients require further studies in larger, clinically well characterized populations, currently underway in our laboratory.

According to this present study, a genetic analysis linked to erythrocyte membrane protein measurements, even among many thousands of SNPs, helped to identify mutations or polymorphisms leading to impaired expression of the ABCG2 protein. These results may promote the understanding of individual pharmacokinetics of ABCG2 substrate drugs or drug derivatives, and a correction of ABCG2 membrane expression by the application of clinically approved agents may provide a tool for personalized drug therapies. These studies also suggest that genetic analysis linked to erythrocyte membrane protein expression may be generally applied to uncover clinically important mutations in complex diseases.

## Methods

### Flow cytometry studies in human erythrocytes

Blood samples obtained from 64 gout and/or hyperuricemic patients and 37 age-matched controls were included in red blood cell expression measurements. Uric acid levels and standard blood parameters were also determined in these patients (National Institute of Rheumatology and Physiotherapy). From the 64 patients, 47 were diagnosed with gout and 17 had significantly elevated serum urate levels. The urate levels were significantly above the normal range in all the gout and hyperuricemic patients examined. Additionally, 127 control samples were collected from healthy volunteers. The research was approved by the Hungarian ethics committee, ETT, and all methods were performed in accordance with the relevant guidelines and regulations. For experiments involving human participants, informed consent was obtained from all participants in this study.

RBC membrane protein determinations were carried out according to our recently developed method^19,20,23^. In brief, we fixed and permeabilized the RBC membranes by using 1% formaldehyde solution, resulting in RBC “ghosts”. Ghosts were incubated with the ABCG2-specific primary antibody (BXP-34, mouse monoclonal antibody, Abcam, cat. ab3379) followed by a secondary, Alexa Fluor 488-labeled goat anti-mouse (H + L) antibody (Thermo Fisher, A-11001), in 96 well plates. The cells were separated from debris on the basis of WGA-Alexa Fluor 647 (Thermo Fisher, cat. W32466) staining. Cellular fluorescence was measured by FACSCanto II flow cytometer equipped with plate loader. Statistical analysis was carried out with STATISTICA 13.1 software (Dell).

### Genetic analysis and vector constructs

Genomic DNA was purified from 300 µL of EDTA-anticoagulated blood samples with Puregene Blood Kit (Qiagen). Sanger sequencing of the *ABCG2* coding region and exon-intron boundaries (exons 2–16) were carried out with primers used by *Zelinski et al*.^49^. TaqMan-based qPCR reactions for SNP detection were performed in a StepOnePlus device (Applied Biosystems) with premade assay mixes (Q141K – cat. 4362691, M71V and R236X - cat. 4351379), or with custom-designed assay mixes (R383C) and master mix (cat. 4371353) from Thermo Fisher. TaqMan probe specificity was verified by sequencing. For expression and transport measurements we used a pIRES2-EGFP bicistronic vector (a kind gift from László Nyitray), pcDNA3.1 (untagged) and pEGFP-C1 (GFP-tagged) vectors (see Suppl. Figure 3), containing wild type ABCG2 and variants (M71V, K86M, Q141K). Site-directed mutagenesis for the M71V variant was carried out in the pcDNA3.1 vector, using standard QuickChange protocol with the following primers:

forward: 5′-CGAATATCAATGGGATC**G**TGAAACCTGGTCTCAAC-3′

reverse: 5′-GTTGAGACCAGGTTTCA**C**GATCCCATTGATATTCG-3′.

Other variants were generated from our previously used vectors by subcloning. For Sf9-baculovirus expression we subcloned these constructs into the pAcUW-21-L plasmid (the ABCG2-Wt, Q141K, K86M in pcDNA vector, and the pAcUW vector was a gift from Csilla Laczka-Ozvegy).

### Mammalian cell culturing, transient transfection, and treatments

HEK293, HeLa and MDCK II. cells were grown in DMEM/high glucose/GlutaMAX medium (Gibco, cat. 10569010) completed with 10% FBS (Gibco, cat. 16140071) and 1% Penicillin-Streptomycin (Gibco, cat. 15070063) at 37 °C (5% CO_2_). Transient transfection of HEK293 and HeLa cells was carried out with Lipofectamine 2000 (Invitrogen, cat. 11668019) in Opti-MEM medium (Gibco, cat. 31985070), whereas MDCKII cells were transfected with Magnetofection Kit (OzBioscences, cat. MTX0750) on Snapwells, according to the manufacturers’ protocol. Drug treatments were carried out 24 h after transfection in completed DMEM medium. The cells were subjected to 1 mM 4-PBA (a gift from András Váradi), 1 µM colchicine (Sigma Aldrich, cat. C9754), or 5 nM bortezomib (PS-341, Calbiochem, CAS 179324-69-7) for 24 h prior to study, whereas 10 µM MG132 (Chalbiochem CAS 133407-82-6) was applied for 4 hours. The efficient inhibitory effect of the applied proteasome inhibitors was ensured by using an *in vitro* proteasome activity fluorometric assay kit (UBPBio, kit J4120).

### Western blotting

Immunoblotting was performed according to standard protocols. Total protein from the cells was precipitated by the addition of a sample buffer containing 0.1 M TRIS-PO_4_, 4% SDS, 4 mM Na-EDTA, 40% glycerol, 0.04% bromphenol blue, and 0.04% β-mercaptoetanol (materials from Sigma-Aldrich) supplemented with a protease inhibitor cocktail (Roche, cat. 11836153001). Red blood cell membrane proteins were prepared according to the protocol described previously^19,56,57^. Proteins were TCA precipitated, washed twice with cold acetone, and resuspended in an SSP buffer (62.5 mM Tris-HCl pH6.8, 2% SDS, 10 mM EDTA-Na, pH = 6.8, 10% glycerol, 0.125 g/ml urea, 0.14 g/ml bromphenol blue, all components from Sigma Aldrich). Equal amounts of proteins were loaded on 10% acrylamide gels. Blots were probed with the following primary antibodies: anti-ABCG2 (BXP-21, Abcam, cat. ab3380), anti-GFP (Abcam, cat. ab290), and anti-β-actin (Sigma, cat. A1978). Goat anti-mouse IgG (H + L) HRP conjugate (Abcam, cat. ab97023) and goat anti-rabbit IgG (H + L) HRP conjugate (Abcam, cat. ab6721) secondary antibodies were used to visualize and quantify the results. Detection was performed with Imobilon Western Chemiluminescent HRP Substrate (Millipore, cat. WBKLS0500) and luminography. Densitometric analysis was performed by ImageJ software v1.42q.

### mRNA expression studies

After 48 h transfection of HEK293 cells with the IRES-Wt-ABCG2, M71V-ABCG2, and Q141K-ABCG2 vectors, we performed mRNA purification according to the manufacturer’s instructions (Purelink RNA MiniKit, Thermo Fisher Scientific, cat. 12183018 A and 12183025). mRNA levels were determined with premade qPCR probes against ABCG2 (Thermo Fisher Scientific, cat. 01053790) and RPLP0 (cat. 99999902), with TaqMan Gene Expression MasterMix (cat. 4369016), according to the manufacturer’s protocol. ABCG2 mRNA levels were normalized to RPLP0 mRNA levels.

### ABCG2 membrane expression studies

We determined the cell surface expression of ABCG2 in transiently transfected HEK293 and HeLa cells 48 h after transfection, and an additional 24 hours after drug treatments. Cells were trypsinized and then the membrane localized ABCG2 was labeled by the 5D3 monoclonal antibody (gift of Bryan Sorrentino, Division of Experimental Hematology, Department of Hematology/Oncology, St. Jude Children’s Research Hospital) by adding 1 µM Ko143 inhibitor (Sigma Aldrich, cat. K2144) to achieve optimal antibody binding. Alexa Fluor 647-labeled IgG2b (Thermo Fisher, cat. A-21242) was used as a secondary antibody. Propidium iodide (final concentration: 1.6 µg/ml, Sigma Aldrich, cat. 4170) was used for separation of the dead cells. Measurements were carried out in a FACS Canto II equipment after gating for live cells.

### Immunofluorescence staining and confocal microscopy

HEK cells were seeded onto 8-well Nunc Lab-Tek II chambered coverglass (Thermo Fisher, cat. 155409) at 5 × 10^4^ cells/well density and grown for 48 h after transfection. For immunostaining of ABCG2, the samples were gently washed with Dulbecco’s modified phosphate-buffered saline (DPBS) and then fixed with 4% paraformaldehyde in DPBS for 10 min at room temperature. After a few washes with DPBS, the cells were further fixed and permeabilized in prechilled (−20 °C) methanol for 5 min. Following further washing steps, the cells were blocked for 1 h at room temperature in DPBS containing 2% bovine serum albumin, 1% fish gelatin, 0.1% Triton-X 100, and 5% goat serum (blocking buffer). The samples were then incubated for 1 h at room temperature with the BXP-21 antibody (Abcam, cat. ab3380) diluted 200x in the blocking buffer. After washing with DPBS, the cells were incubated for 1 h at room temperature with Alexa Fluor 594-conjugated goat anti-mouse IgG (H + L) secondary antibody (Thermo Fisher, cat. A11005), diluted 250x in blocking buffer. Nuclei were stained with DAPI (1 µM final concentration).

MDCKII cells were cultured on the insert of 6-well Snapwell apparatus (Corning, Costar, Cat.No. 3801, clear polyester membrane) until reaching polarization after 6 days. The immunfluorescence staining followed the same protocol as for HEK cells, then the cells were labeled with Bxp-21 and anti-Na^+^/K^+^ -ATPase antibody (Abcam, cat. ab353) diluted 500x, as well as with Alexa Fluor 488-conjugated goat anti-mouse IgG (H + L) (Thermo Fisher, cat. A32723) and Alexa Fluor 594-conjugated goat anti-chicken IgG (H + L) (Thermo Fisher, cat. A11042) secondary antibodies (250x). Following antibody staining, membranes were released from the inserts using a scalpel and placed on glass slides with the apical membrane upwards, then covered with Vectashield HardSet antifade mounting medium (Vector Laboratories, cat. H-1400) and coverslips. The cells were examined under a Ziess LSM 5710 laser scanning fluorescence confocal microscope (40x, oil immersion objective), and images were processed with ZEN 2012 software.

### Hoechst 33342 uptake measurements

Hoechst 33342 (Hst, Thermo Fisher, cat. H1399) uptake was determined in transiently transfected HEK293 cells (with pIRES vectors) 48 h after transfection. Cells were trypsinized and then preincubated for 5 minutes in 37 °C with or without Ko143 inhibitor (2 µM) and then uptake were started with the addition of Hoechst 33342 dye (1 µM). Measurements were carried out in FACS Canto II with continuous sampling for 80 sec (linear phase of the uptake). Then GFP-positive cells were gated for 20 second intervals. Initial slopes were calculated from these five points (0 sec to 80 sec) by linear fitting (see Supplementary Figure 11).

### Sf9 membrane preparation and ATPase activity

Sf9 cells were grown as a suspension culture at 27 °C in TNM-FH medium (Sigma-Aldrich, cat. T3285) supplemented with 5% FBS. For ABCG2 expression in Sf9 cells, the cells were infected by flashBAC ULTRA™ Kit (Oxford Expression Technologies Ltd.) based on the manufacturer’s protocol. Membrane preparation was carried out according to Telbisz *et al*.^58^.

ATPase activity was measured in isolated membranes of Sf9 cells expressing ABCG2 wild type or its mutant variants by colorimetric detection of inorganic phosphate liberation, as previously described^32^. Figures represent the mean values of at least three independent experiments performed in duplicates, measured in two different membrane preparations for each variant of ABCG2. ATPase activity of ABCG2 in proteoliposomes was determined as described previously^34,58^. In brief, the measurement of ATPase activity was started by the addition of 2 mM MgATP and terminated after 20 min with 2% (w/v) SDS. Inorganic phosphate was determined by colorimetric reaction. Cholesterol loading of the membranes was achieved by the addition of 0.6 mM RAMEB-cholesterol (Cyclolab Ltd, Budapest, Hungary) and incubation for 10 min on ice according to Telbisz *et al*.^34^. We used 10 µM quercetin, since quercetin gives maximum stimulating effect of the ABCG2-ATPase activity in a range of 1–100 µM, according to our previously published measurements^59^.

### In silico methodologies

#### Structures

the ABCG2 NBD (residues 31–304) structure was dissected from our ABCG5/ABCG8 homology model^35^. Details and validity are described in the Supplementary material. The NBD with the M71V mutation was generated using VMD^60^.

#### Molecular dynamics (MD) simulations

Three 100 ns long MD simulations were performed with the wild type and mutant NBDs using GROMACS with CHARMM36m force field^61,62^. For simulation details see the Supplementary materials. Analysis was performed by employing GROMACS tools, VMD Network Wizard Plugin, and the MDAnalysis Python package^63^.

### Data availability

The datasets generated during and/or analysed during the current study are available from the corresponding author on reasonable request.

## Electronic supplementary material

Supplementary information
