# Supplementary material for: Clinically relevant mutations in the ABCG2 transporter uncovered by genetic analysis linked to erythrocyte membrane protein expression
Source: Sci Rep. 2018 May 10;8:7487. doi: 10.1038/s41598-018-25695-z (PMC5945641; doi:10.1038/s41598-018-25695-z)

Supplementary Information

for

"Clinically relevant mutations in the ABCG2 transporter uncovered by genetic analysis linked to erythrocyte membrane protein expression"

by

Boglárka Zámbó, Zsuzsa Bartos, Orsolya Mózner, Edit Szabó, György Várady, Gyula Poór, Márton Pálinkás, Hajnalka Andrikovics, Tamás Hegedűs, László Homolya, and Balázs Sarkadi

Supplementary methods

In silico methods

Structures. ABCG2 NBD (a.a. 31-304) structure was dissected from our ABCG5/ABCG8 homology model[29]. Although the structure of ABCG2 has been recently determined experimentally, the resolution in the cytosolic regions is low[40]. The NBD of this published cryo-EM structure could not be modeled *de novo*, but was built using homology modeling based on the ABCG5/ABCG8 structure, and many side chains could not be assigned. Our homology model is similar to the cryo-EM structure and, because of the homology between ABCG5/ABCG8 and ABCG2, the side chains are the same in many cases. The RMSD (root mean square deviation) of our model from cryo-EM model is low, 1.8Å.

Molecular dynamics simulations. The input files for all steps (energy minimization, equilibration, and production run) were generated by the CHARMM-GUI web interface[51,52]. The following options were set: terminal residues were patched by ACE (acetylated N-terminus) and CT3 (N-methylamide C-terminus), 150 mM KCl in TIP3 water were used, grid information for PME (Particle-Mesh Ewald) electrostatics was generated automatically, and a temperature of 310 K was set. The two structures were energy minimized using the steepest descent integrator (maximum number to integrate: 50,000 or converged when force is <1,000 kJ/mol/nm). From the energy minimized structures we forked 3-3 parallel NVT equilibrium simulations followed by 100 ns long production runs. Nose-Hoover thermostate and Parrinello-Rahman barostat were applied. Electrostatic interactions were calculated using the fast smooth PME algorithm, and LINCS algorithm was used to constrain bonds. Constant particle number, pressure, and temperature ensembles with a time step of 2 fs were used. Simulations were executed on a GPU cluster of the NIIF National Information Infrastructure Development Institute

(http://www.niif.hu/en) and Wigner GPU Laboratory (http://gpu.wigner.mta.hu). The systems reached equilibrium in the first 20 ns of MD simulations, thus the last 80 ns parts were used in the analysis.

Network analysis. Since it is difficult to achieve a sufficient time scale in MD simulations to directly describe molecular events happening on the millisecond to minute time scale, statistical methods are used to infer altered dynamics occurring at longer time scales. We used VMD NetworkView[57] supplemented with Carma[58] to calculate correlation in motion of amino acids, to build a network using amino acids as nodes and pairwise correlations as weighted edges, to detect communities by the Girvan-Newman algorithm[59], and to visualize the network mapped to the structure.

Supplementary Figures

Supplementary Figure 1: *ABCG2 protein membrane topology and the position of the most relevant mutations and polymorphisms.*

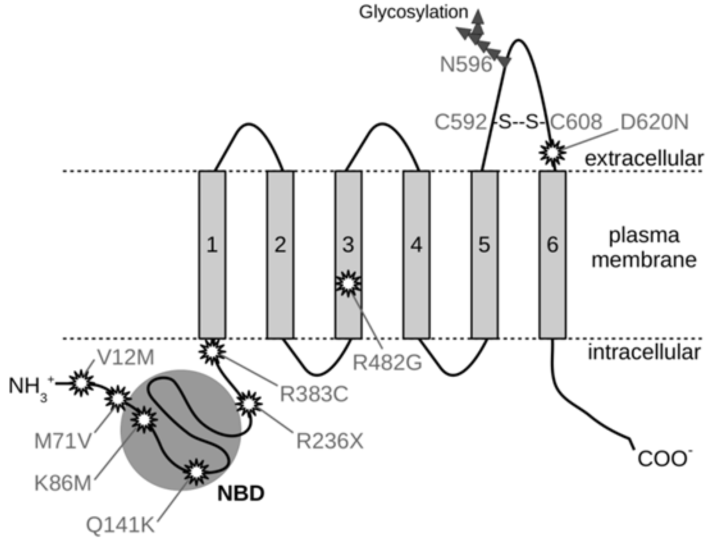

Supplementary Figure 2: *Western blot analysis of red blood cell membranes.* Red blood cell membranes were prepared from individuals possessing wild type ABCG2 or carrying the M71V mutation. The Western blot was developed with the ABCG2-specific BXP-21 monoclonal antibody (see Methods).

Supplementary Figure 2:

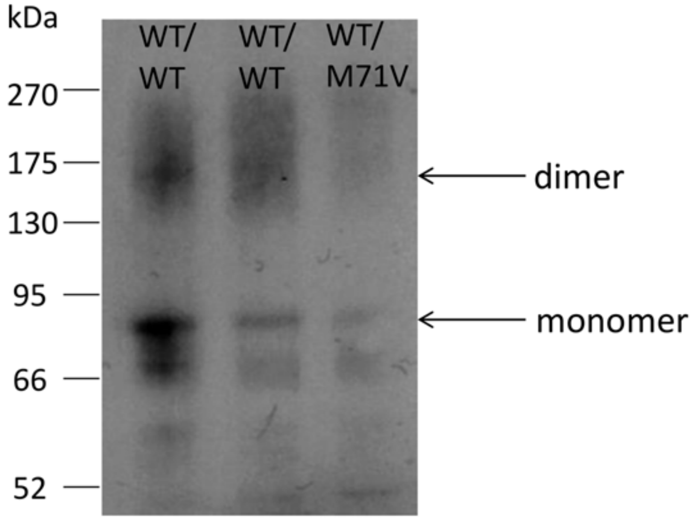

Supplementary Figure 3: Three types of ABCG2 expression vector constructs were used in this study. For direct visualization of the ABCG2 protein quantity and localization, we expressed an N-terminally GFP-tagged ABCG2 protein, by using a GFP-ABCG2 fusion construct in pEGFP-C1 type vector (left panel). For the expression of the untagged ABCG2 protein we used the pcDNA3.1-ABCG2 vector (mid panel). For a parallel determination of ABCG2 expression and transfection efficiency, and also for using flow cytometry gating based on GFP expression, we used the pIRES2 type vector (right panel). In this case GFP and ABCG2 are expressed in a coupled way in the transfected cells (figures were generated with SnapGene1.1.3 software.)

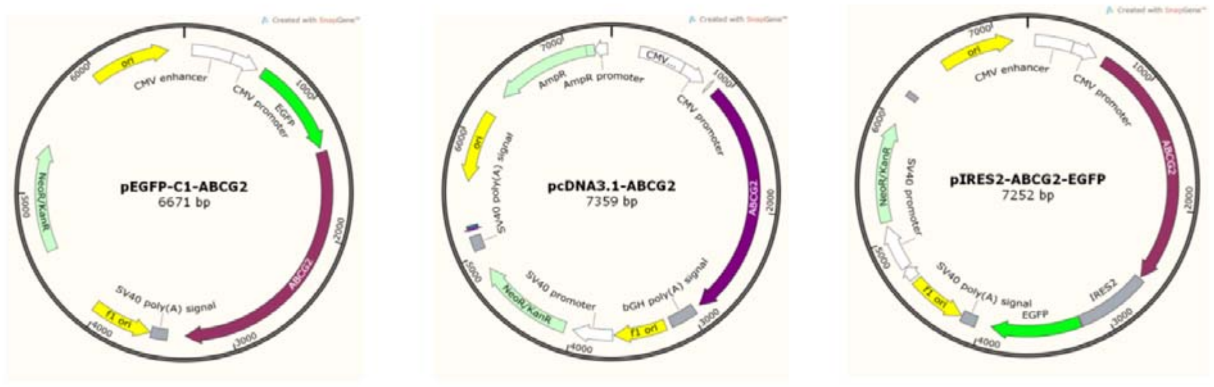

Supplementary Figure 4: *mRNA expression levels of transiently transfected HEK293 cells with IRES-type vectors.* After 48 hours transfection we did not observe differences between ABCG2 mRNA expressions in the case of the wt, M71V and Q141K ABCG2 constructs. (ns. indicates no significant differences)

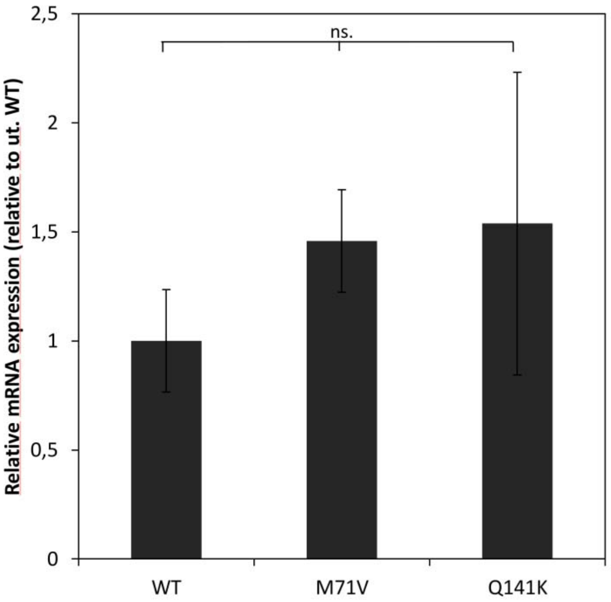

Supplementary Figure 5: *ABCG2 protein expression in various mamalian cell lines by using different expression vectors.*

Panels a) and b**)** Western blotting of cell extracts from HEK293 and HeLa cells, respectively, transfected with vectors expressing the ABCG2 wild type and the ABCG2-M71V proteins, or these proteins with an N-terminal GFP-tag.

c) Expression levels of the ABCG2 wild type and the ABCG2-M71V variants in HEK293 and HeLa cells, based on band intensities in Western blots, normalized to beta actin. The results are presented as relative expression levels, compared to that of the ABCG2-WT. The different proteins examined in the Western blot were developed by the respective antibodies and cropped from the indicated parts of the same gel and blot.

d) Cell surface expression levels of the ABCG2 wild type and the ABCG2-M71V variants in HEK293 and HeLa cells, determined by 5D3 antibody binding and flow cytometry.

Supplementary Figure 5.

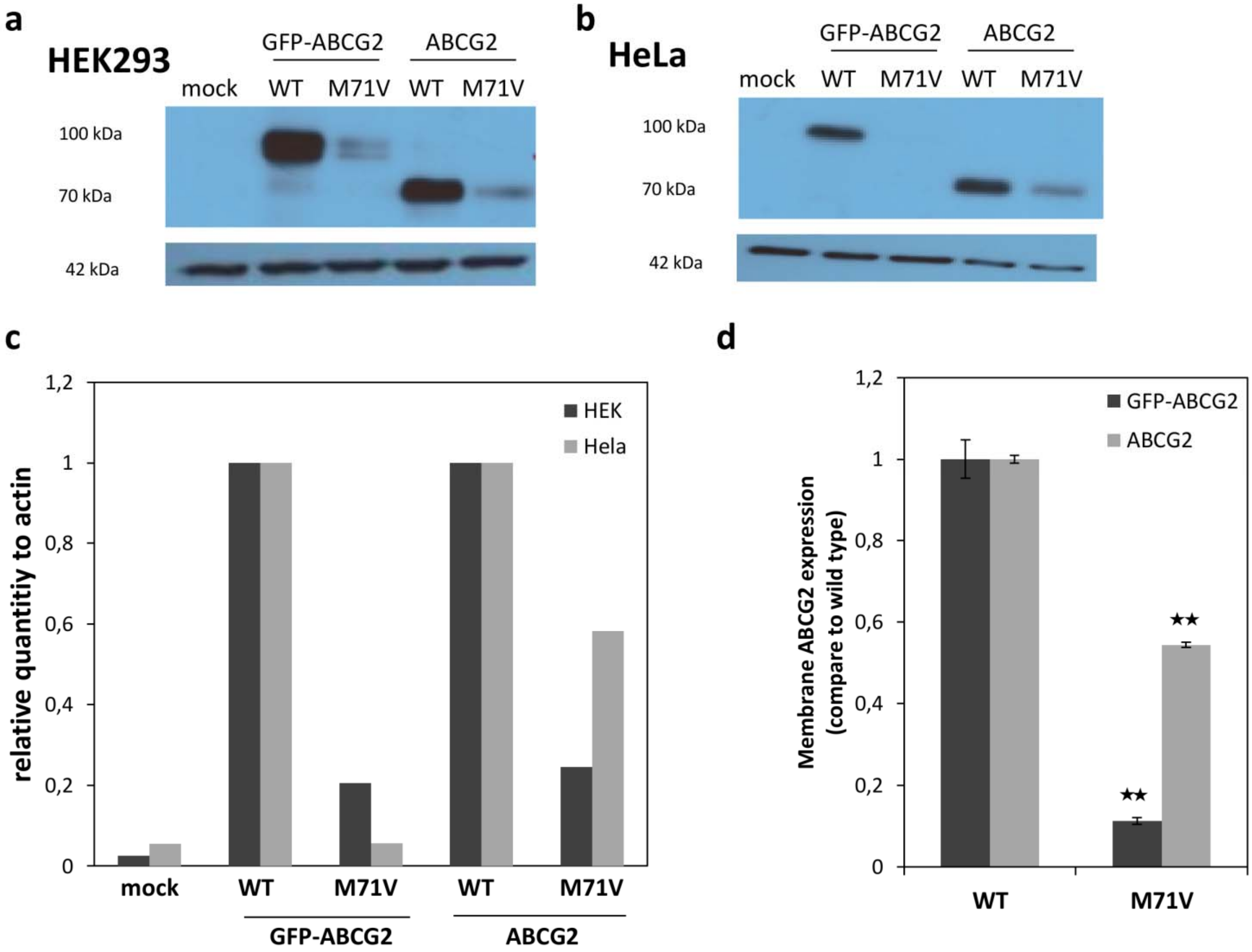

Supplementary Figure 6: *Quercetin and uric acid stimulate ABCG2-ATPase activity of the human ABCG2-Q141K and ABCG2-M71V proteins, expressed in Sf9 insect cells.*

In isolated Sf9 cell membrane vesicles, containing either the human wild-type ABCG2, the ABCG2-Q141K, or the ABCG2-M71V protein, ABCG2-ATPase activity was significantly stimulated by quercetin and by uric acid. Vanadate-sensitive ATPase activity was measured in cholesterol-loaded membrane vesicles (see Material and Methods), with the reaction conditions: 10 µg membrane protein, 3.3 mM ATP, incubated at 37 °C for 20 min, in the presence of 5 µM quercetin or 5 mM uric acid (* indicates $p<0.05$, ** indicates $p<0.01$, in two parallels). Quercetin is a well described activator of the ABCG2-ATPase in the range of 1-100 uM. Uric acid had a maximum stimulatory effect in concentrations between 2-5 mM (data not shown). The ABCG2-K86M catalytic mutant variant has no ATPase activity either in the absence or presence of the activators.

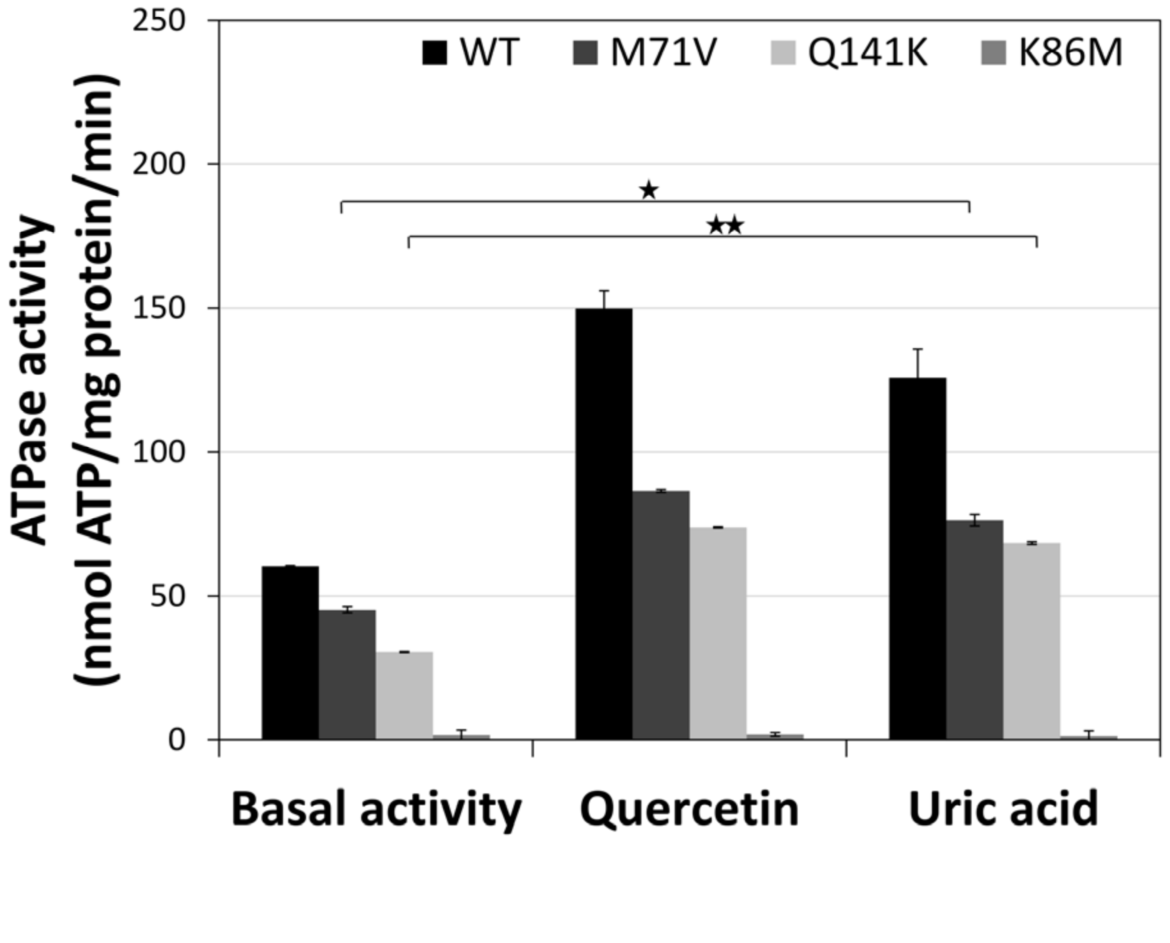

Supplementary Figure 7: *Val at position 71 exhibits altered interactions with amino acids within the ABCG2-NBD.* Pairwise amino acid contacts (distance of Cα < 7.5Å) were counted in frames between 20-100 ns of the MD trajectory. Values for amino acids interacting with residue 71 over 50% of the frames either in the Wt or M71V NBD were selected and plotted. See Fig. 4 of the main manuscript for their spatial localization.

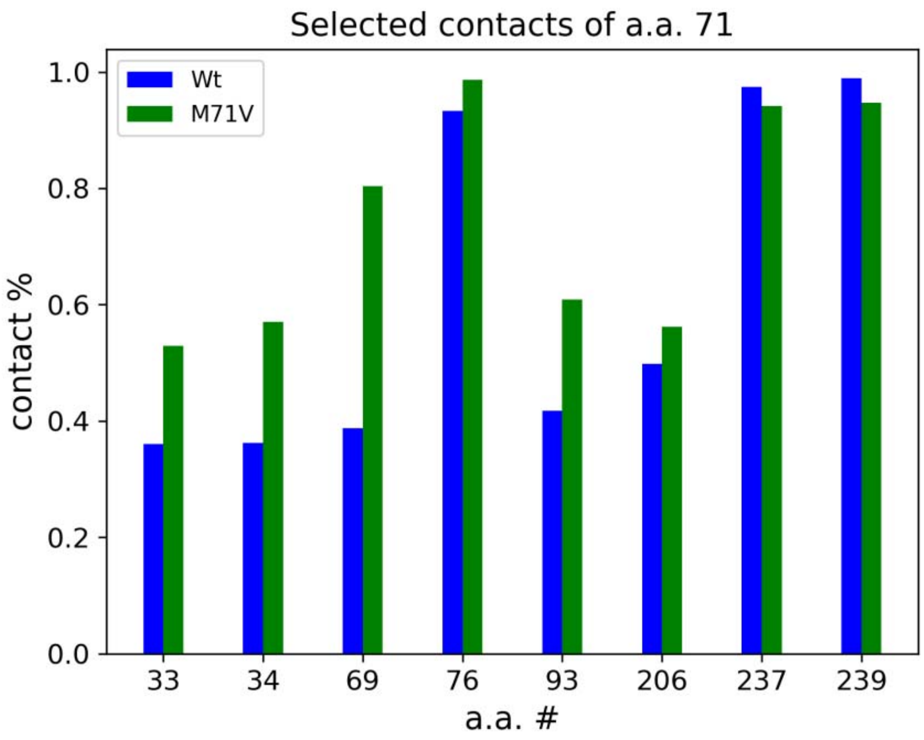

Supplementary Figure 8: *Impact of various compounds on ABCG2 expression in HEK293 cells (original blot of the details presented in Figure 5A of the main manuscript).* Three different types of antibodies (ABCG2/BXP-21, anti-actin and anti GFP – see Methods) were used, and the blot was developed repeatedly.

Labels on the original blot: vector c.: vector control, ut: untreated, 4-PBA: 4-phenylbutyrate, Chn: colchicine, mock tr.: mock (DMSO) treated samples.

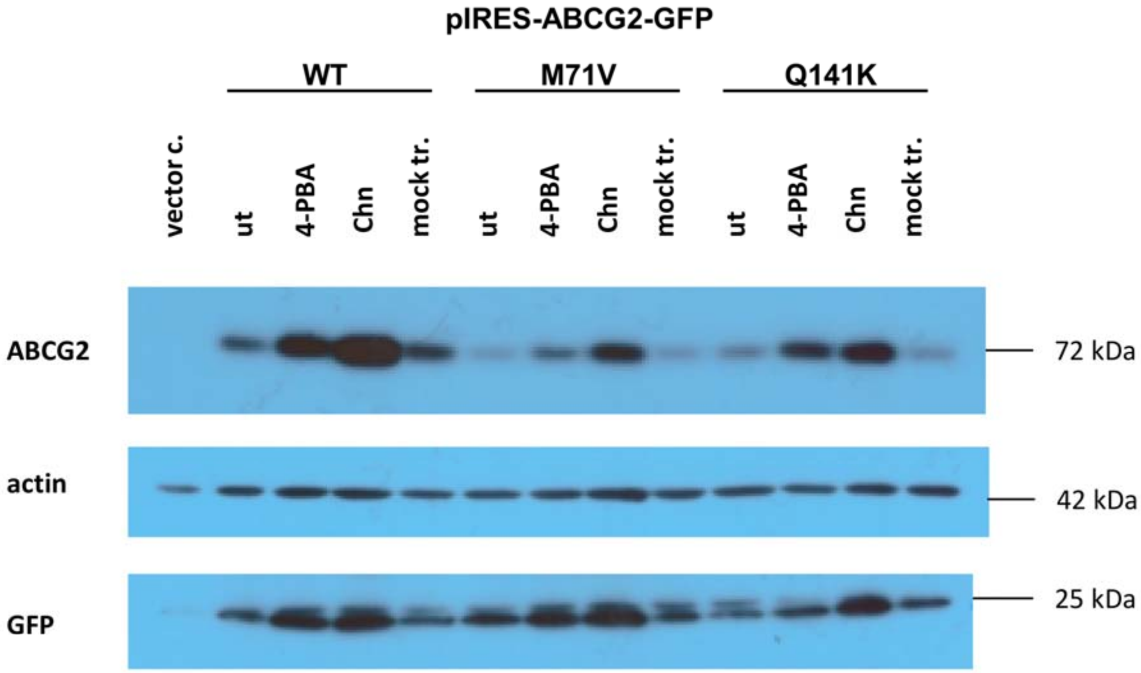

Supplementary Figure 9: Effect of 4-PBA and colchicine on the expression of ABCG2, GFP, and actin. Western-blot analysis demonstrates a marked increase in ABCG2 expression level in response to 4-PBA and colchicine (Chn), whereas GFP expression was elevated to a lesser extent. The change in actin expression was negligible. Cells were treated with 1 mM 4-PBA and 1 μM colchicine for 24 h prior to the experiments (n=3, mean±SEM).

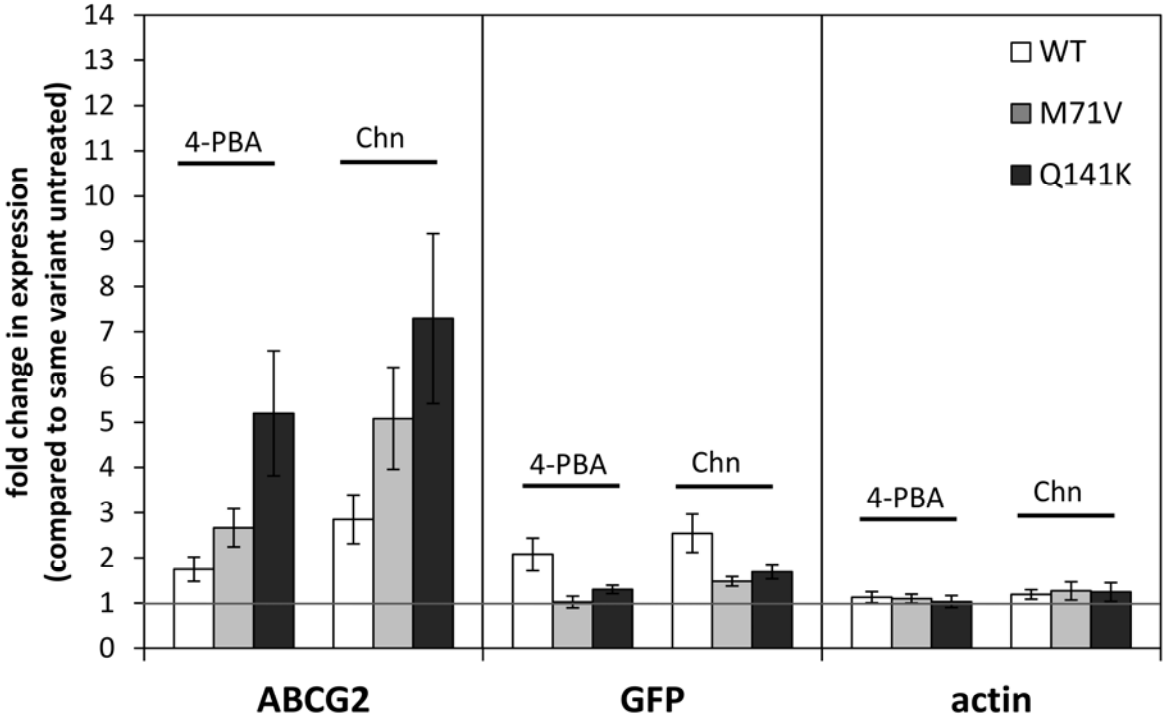

Supplementary Figure 10: *Effects of proteasome inhibitors on the surface expression of the Wt and M71V and Q141K variants of ABCG2.* 5D3 labeling and flow cytometry were carried out following a 24 h pretreatment with 5 nM bortezomib (bzb) or after a 4 h pretreatment with 10 μM MG132. ut.: untreated cells.

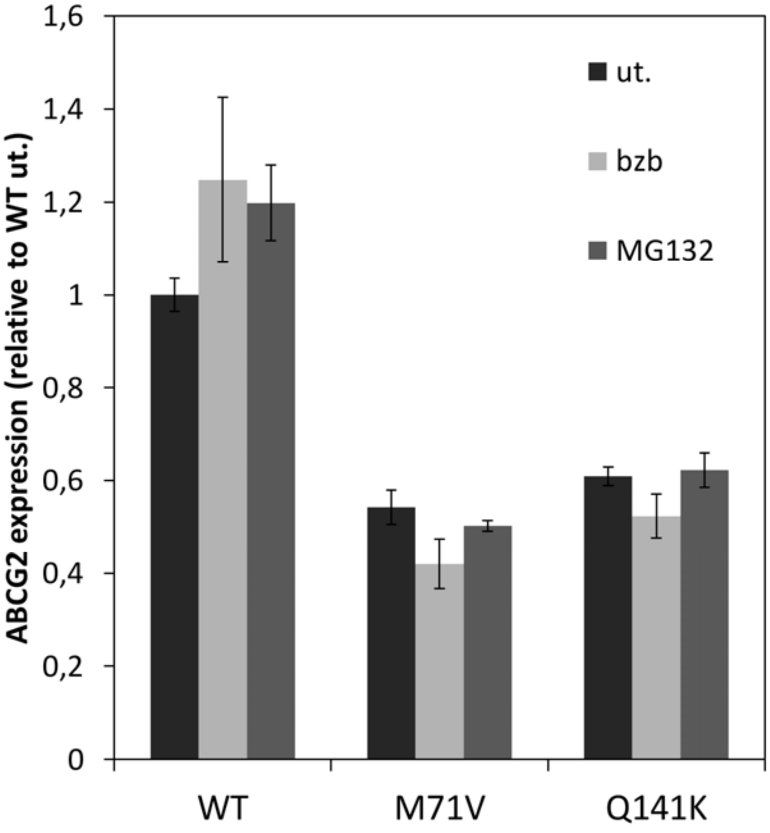

Supplementary Figure 11*: Kinetics of fluorescent Hoechst 33342 dye accumulation in HEK293 cells expressing the ABCG2 variants, measured by flow cytometry*.

HEK293 cells expressing the ABCG2-WT, ABCG2-M71V, ABCG2-Q141K or ABCG2-K86M protein were incubated with 1 μM of Hoechst 33342 (Hst). Solid lines show the accumulation of cellular, DNA-bound Hst dye, based on fluorescence intensity in ABCG2 expressing cells. The dashed lines show data obtained in cells treated with Hst, and with the ABCG2 specific inhibitor Ko143 (2 μM). For initial slope parameters see Figure 3.A, in the main manuscript.

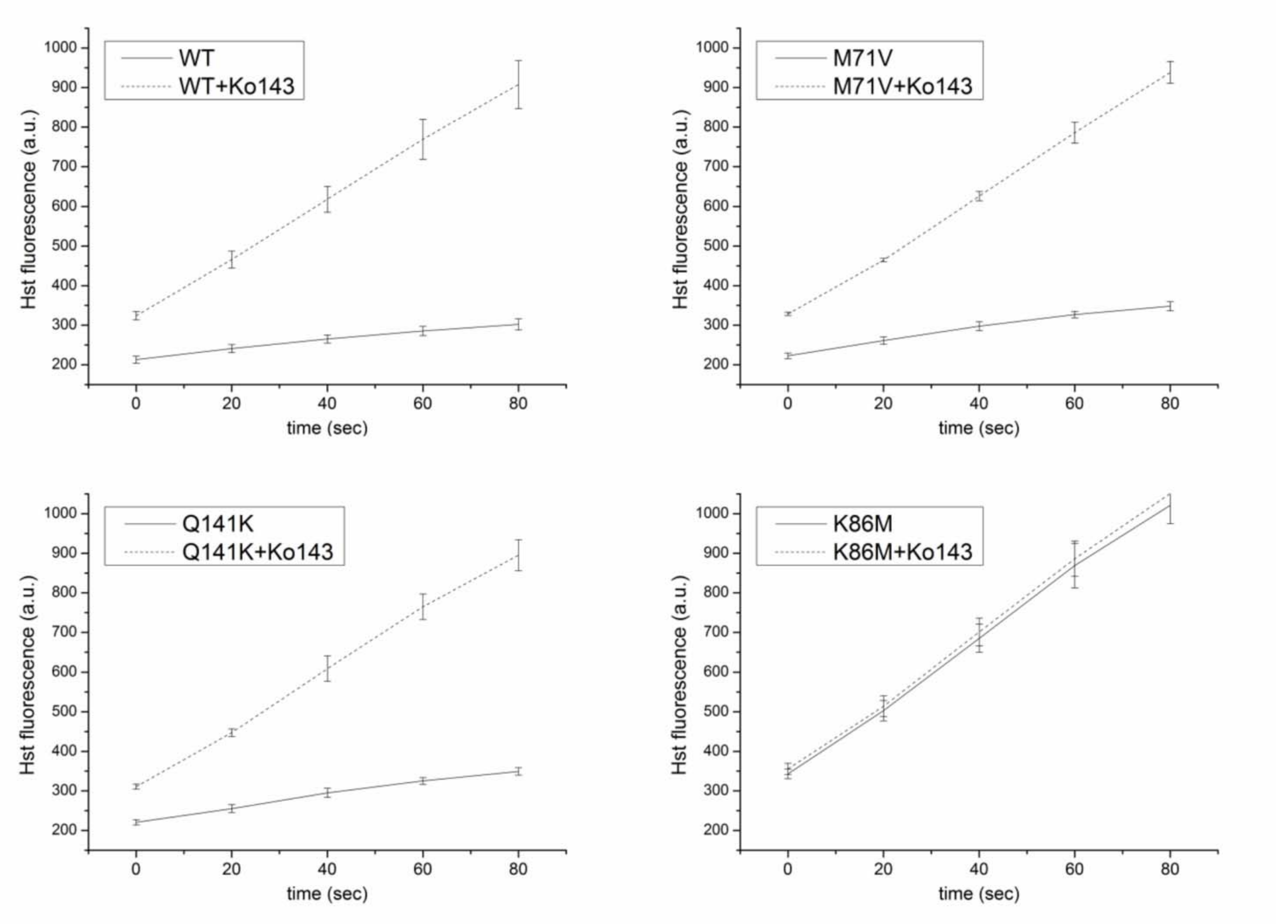

Supplement: Supplementary file 1 — Supplementary information [file 41598_2018_25695_MOESM1_ESM.pdf]
